# Artificial intelligence-empowered 4D bioprinting for craniomaxillofacial subunit reconstruction: a comprehensive review

**DOI:** 10.3389/fbioe.2026.1964537

**Published:** 2026-10-20

**Authors:** Hailian Chen, Shuang Zou, Jianping Song

**Affiliations:** 1 Department of Nursing, The Second Affiliated Hospital of Zhejiang University School of Medicine, Hangzhou, China; 2 Zhejiang University, Hangzhou, China

**Keywords:** 4D bioprinting, artificial intelligence, craniomaxillofacial subunit reconstruction, facial vascularized composite allotransplantation, reconstructive surgery

## Abstract

Craniomaxillofacial subunit transplantation represents one of the most technically demanding reconstructive strategies for repairing anatomical subunit defects involving the cranium, mandible, maxilla, orbital cavity and nose. Conventional reconstructive approaches are hindered by inherent limitations in personalized anatomical matching, dynamic tissue adaptability, and long-term therapeutic efficacy. The integration of artificial intelligence (AI) and four-dimensional (4D) bioprinting has revolutionized craniomaxillofacial reconstructive surgery. This review summarizes state-of-the-art advances in AI-enabled 4D bioprinting for craniomaxillofacial subunit repair, covering core application scenarios including bone defect regeneration, facial soft tissue and cutaneous organ reconstruction, peripheral neural regeneration, and vascularized composite tissue fabrication. We elaborate the synergistic potential of AI and 4D bioprinting in craniomaxillofacial subunit reconstruction, and dissect critical bottlenecks including inadequate material performance, low precision of shape deformation programming, and limited clinical translatability of existing AI models. This work provides valuable insights for further translational research of AI-integrated 4D bioprinting in craniomaxillofacial subunit reconstruction.

## 1 Introduction

The craniomaxillofacial region features intricate anatomical architectures, rigorous aesthetic requirements and sophisticated functional demands, making defect reconstruction one of the greatest challenges in reconstructive surgery (Gu et al., 2023). Traditional reconstructive strategies are plagued by donor site morbidity, limited tissue availability, and poor morphological and functional matching (Eber et al., 2025). As an innovative reconstructive paradigm, craniomaxillofacial subunit transplantation (CST) achieves superior integrated recovery of function and aesthetics compared with conventional craniomaxillofacial repair techniques (Tchiloemba et al., 2021). Also termed facial vascularized composite allotransplantation (fVCA), CST relies on *en bloc* transplantation of compound tissues containing skin, mucosa, blood vessels, muscles and bone to realize homologous tissue replacement (Ren et al., 2024). Nevertheless, widespread clinical translation of CST is restricted by fundamental barriers. Klimitz et al. (2025) demonstrated that although subunit transplantation is anatomically feasible, the intensity of immunosuppressive regimens is comparable to full-face transplantation, and lifelong administration of immunosuppressants induces severe adverse effects. Immune rejection and donor shortage remain two dominant obstacles limiting routine clinical application of CST.

Tissue engineering and regenerative medicine integrate multidisciplinary theories and technologies from biology, engineering and materials science to fabricate functional living constructs with biomaterials, seed cells and growth factors for defect repair and tissue function restoration, circumventing immune rejection and donor scarcity (Ye et al., 2026). Four-dimensional (4D) bioprinting emerges as a transformative technology in tissue engineering and regenerative medicine by incorporating time as the fourth dimension. Printed constructs undergo preprogrammed dynamic shifts in morphology or functionality when exposed to external stimuli such as temperature, humidity, pH or magnetic fields (Roshith, 2026). Such adaptive deformation enables minimally invasive delivery of 4D bioprinted scaffolds, which subsequently conform to irregular defect contours, dynamically modulate mechanical microenvironments, and guide oriented regeneration of host tissues, opening new avenues for CST (Guo et al., 2024).

While conventional three-dimensional (3D) bioprinting techniques, including stereolithography (SLA) and digital light processing (DLP), as well as their derivative 4D-printing technologies, enable the fabrication of constructs with micrometre-scale resolution and sophisticated architectures (Milton et al., 2023), the structural design of conventional 4D-printed constructs relies heavily on empirical knowledge and iterative trial-and-error to achieve material distributions that yield target deformation upon specific stimuli (Chen et al., 2026). Furthermore, substantial challenges remain in efficiently screening combinatorial solutions tailored to individualised and heterogeneous physiological microenvironments within the large parameter space spanned by material formulations, structural variables and deformation kinetics (Akhoundi, 2025). Pre-programmed deformation features of such constructs also complicate the prediction of spatiotemporal deformation profiles and the implementation of controllable programming. Under such circumstances, artificial intelligence (AI) serves as a powerful auxiliary tool that leverages AI algorithms to deduce optimal initial material distributions and structural parameters across a high-dimensional design space, thereby accelerating the optimization process and enabling efficient, high-precision inverse design of 4D-printed structures (Kang et al., 2026; Chiu et al., 2025; Chen et al., 2025). This AI-empowered strategy can be synergistically integrated with established bioprinting platforms, offering a viable route toward engineering smart functional 4D-printed constructs capable of dynamic response and adaptation to physiological microenvironments.

Against this background, the present review concentrates on cutting-edge progress of AI-empowered 4D bioprinting for craniomaxillofacial subunit reconstruction. We summarize the methodologies and applications of AI-assisted 4D bioprinting in craniomaxillofacial regenerative reconstruction, aiming to offer comprehensive references for cross-disciplinary research in this field.

## 2 AI-empowered 4D bioprinting: fundamental mechanisms and intelligent design frameworks

### 2.1 Technical fundamentals

Derived from three-dimensional (3D) additive manufacturing, 4D bioprinting introduces time as the fourth dimension to realize preprogrammed dynamic shifts in shape, mechanical properties or biological functionality upon external stimulation, with its core built on the integration of smart materials and additive manufacturing (Guo et al., 2024). Four major categories of smart materials are widely adopted for craniomaxillofacial 4D bioprinting: shape memory polymers (SMPs), shape memory alloys (SMAs), hydrogels, and cell-laden bioinks.

Shape memory polymers (SMPs) possess tunable glass transition temperatures, adjustable elastic moduli and modifiable degradability, rendering them ideal candidates for fabricating nerve-guiding conduits and adaptive conformal shells for non-load-bearing regions (orbital wall, zygomatic body, anterior maxillary sinus wall) (Song et al., 2025). The elastic moduli of SMPs typically range from approximately 0.1–100 MPa, enabling mechanical matching with native non-load-bearing craniofacial tissues such as trabecular bone (0.1–2 GPa) (Gu et al., 2023) and facial skin (0.1–10 MPa) (Ferrer et al., 2024).

Based on their compressive strength (>800 MPa), elastic modulus (100–110 GPa) and biocompatibility, titanium and its alloys are regarded as the clinical gold-standard materials for load-bearing craniomaxillofacial reconstruction (Gan et al., 2025). However, conventional titanium alloys are inert fixation materials and cannot provide dynamic mechanical modulation (Ferrer et al., 2024). Nickel-titanium-based shape memory alloys (SMAs) possess additional superelasticity and robust shape recovery capacity on the basis of titanium alloys. Relying on the martensitic phase-transformation mechanism, they can realize active shape restoration at body temperature, which renders them applicable for active distractors and deep fixation screws in mandibular distraction osteogenesis (Ahmed et al., 2026). However, these alloys are non-degradability and suffer from potential nickel ion leakage, thereby limiting their long-term *in vivo* retention (Es-Souni et al., 2005). Consequently, extensive research efforts are currently focused on developing advanced surface modification strategies, such as anodization and composite coatings, to mitigate nickel ion release and enhance the biocompatibility of NiTi implants (Korkmaz and Sağlam, 2022; Ohtsu et al., 2025; Safavi et al., 2023).

Hydrogels feature ultra-low elastic moduli, typically ranging from 0.2 to 100 kPa (Cianciosi et al., 2023; Gu et al., 2023), which closely match the stiffness of native brain (0.5–1 kPa), liver (0.65–2 kPa), and adipose tissue (1.6–2.9 kPa) (Guimarães et al., 2020). Consequently, these materials are predominantly applied in soft tissue engineering rather than load-bearing craniofacial reconstruction. This mechanical property renders hydrogels particularly suitable for repairing soft tissue subunits, including facial skin, oral mucosa, and peripheral nerves. 4D-bioprinted hydrogel expanders have been successfully employed to generate autologous skin flaps for auricular and nasal reconstruction (Wang et al., 2026).

Cell-laden bioinks, composed of hydrogel matrices, living cells and bioactive additives, serve as core carriers for 4D bioprinting to fabricate time-evolving cell-based constructs including bone, cartilage and vascularized tissues (Lai et al., 2024). Consistent with their hydrogel matrix composition, these bioinks exhibit low elastic moduli (typically in the kPa range), which are conducive to cell viability, migration, and tissue morphogenesis during early-stage culture (Cianciosi et al., 2023). The formed constructs acquire tissue-specific mechanical properties through subsequent cellular remodeling and extracellular matrix deposition. This cell-driven maturation mechanism lays the foundation for the application of 4D-bioprinted constructs in cranio-maxillofacial tissue engineering. The key mechanical properties and craniomaxillofacial applications of these four material categories are summarized in Table 1.

**TABLE 1:** Mechanical properties and applications of smart materials for craniomaxillofacial 4D bioprinting.

Material category	Elastic modulus	Key features	Matched tissues	Applications	Major limitations
Shape memory polymers (SMPs)	∼0.1–100 MPa	Tunable elastic modulus; shape memory effect	Trabecular bone (0.1–2 GPa); facial skin (0.1–10 MPa)	Nerve-guiding conduits; conformal shells for orbital wall, zygomatic body, anterior maxillary sinus wall	Compressive modulus <100 MPa; insufficient for occlusal load-bearing
Titanium alloys	100–110 GPa (compressive strength >800 MPa)	High strength; excellent biocompatibility	Cortical bone (10–20 GPa)	Load-bearing reconstruction (mandible, craniofacial osteosynthesis)	Inert fixation; no dynamic mechanical modulation
NiTi shape memory alloys (SMAs)	∼100 GPa	Superelasticity; shape recovery (martensitic phase)	Cortical bone (10–20 GPa)	Active distractors and deep fixation screws in mandibular distraction osteogenesis	Non-degradable; potential nickel ion leakage
Hydrogels/cell-laden bioinks	0.2–100 kPa	Ultra-low elastic modulus; high water content; cytocompatible	Brain (0.5–1 kPa); liver (0.65–2 kPa); adipose tissue (1.6–2.9 kPa)	Soft tissue filling; skin flap expansion; vascularized tissue constructs	No mechanical loading; limited to soft tissue

4D, four-dimensional; GPa, gigapascal; kPa, kilopascal; MPa, megapascal; NiTi, nickel-titanium; SMA, shape memory alloy; SMP, shape memory polymer.

The properties of 4D printed smart materials lay a technical foundation for minimally invasive and personalized reconstruction in craniofacial subunit repair. Nevertheless, the nonlinear constitutive relation and shape memory effect of smart materials are highly dependent on temperature, stress history and deformation rate, making it difficult to accurately predict their deformation behaviors (Bond et al., 2023). Furthermore, craniofacial anatomical structures exhibit significant inter-individual variations, so a single material formulation or printing strategy cannot accommodate all clinical scenarios. Conventional 4D printing struggles to meet the urgent demands for rapid response and high repeatability required by individualized precision medicine. The introduction of AI offers a systematic solution to the above bottlenecks. It empowers 4D printing from three dimensions: material screening, manufacturing path optimization and deformation programming, facilitating the intelligent design and fabrication of implants. To systematically elaborate three core AI technical frameworks addressing the above bottlenecks of conventional 4D bioprinting, we summarize the fundamental mechanisms, representative algorithms, experimental achievements and craniomaxillofacial translational value of AI-enabled 4D bioprinting in Table 2.

**TABLE 2:** AI-empowered 4D bioprinting: fundamental mechanisms and intelligent design frameworks.

AI empowerment dimension	AI methods	Key outcomes	Craniomaxillofacial relevance
AI-accelerated intelligent biomaterials	SVR, XGB, GPR, ANN, PIANN	GPR: optimized tBA/HDDA microneedles; 10-day wound healing (Lee et al., 2026); ANN: UTS/shape memory prediction error reduced 40%–50% (Kumar et al., 2026); PIANN: 2-curve training predicts stress/free strain (Yan et al., 2025)	Personalized SMP screening; data-driven mechanical/shape property regulation
AI-optimized processing parameters	DRL-PN	Path generation efficiency improved by 99.8%; structural deformation reduced by 64.0%; residual stress reduced by 21.8% (Bai et al., 2025)	Precision manufacturing; multi-material co-printing
AI-mediated inverse design and deformation programming	FCNs	IoU >90% (Chiu et al., 2025); depth deviation <10% (Chen et al., 2025)	Patient-specific facial subunit reconstruction

4D, four-dimensional; AI, artificial intelligence; ANN, artificial neural network; DRL-PN, deep reinforcement learning-based path optimization; FCN, fully convolutional network; GPR, gaussian process regression; IoU, intersection over union; PIANN, physics-informed artificial neural network; SMP, shape memory polymer; SVR, support vector regression; UTS, ultimate tensile strength; XGB, extreme gradient boosting.

### 2.2 AI accelerates the development of intelligent biomaterials

Shape memory polymers (SMPs) performance is highly coupled with material composition, structural parameters, and fabrication conditions. Traditional experimental strategies are inefficient and incapable of precise property regulation and optimal parameter screening. In contrast, AI and data-driven modeling enable accurate performance prediction using limited experimental data, effectively reducing experimental costs and accelerating the iterative optimization of smart biomaterials.

Lee et al. (2026) developed thermally responsive SMPs copolymers based on tert-butyl acrylate (tBA) and 1,6-hexanediol diacrylate (HDDA). Multiple machine learning models including support vector regression (SVR), extreme gradient boosting (XGB) and Gaussian process regression (GPR) were established to quantify correlations between material formulations and shape memory recovery performance. High-precision GPR surrogate models rapidly identified optimal processing parameters to fabricate thermally responsive biomimetic microneedle patches. *In vivo* evaluation in diabetic rodent models confirmed that the optimized patches significantly accelerated wound healing, achieving near-complete closure within 10 days with enhanced re-epithelialization and collagen remodeling (Lee et al., 2026). Kumar et al. (2026) systematically compared multiple machine learning algorithms for predicting the properties of 4D-printed polyurethane/Eudragit® S100/metronidazole filaments. Their work demonstrated that the artificial neural network (ANN) achieved the highest predictive accuracy, enabling reliable forecasts of ultimate tensile strength and shape memory activation behavior. The ANN model reduced prediction errors by approximately 40–50% compared to conventional linear regression and Least Absolute Shrinkage and Selection Operator (LASSO) methods, underscoring the superiority of data-driven approaches for tailoring the mechanical performance of 4D printed SMPs. Yan et al. (2025) developed a physics-informed artificial neural network (PIANN) for amorphous SMPs that requires only two temperature-stress curves for training. Despite the minimal data requirement, their PIANN accurately predicted stress evolution during thermal programming and stress recovery after both cold and hot programming. Moreover, the model successfully predicted free strain responses upon heating, demonstrating its strong generalization capability. This approach offers a promising pathway to overcome the scarcity of large experimental datasets, a critical barrier for AI adoption in craniomaxillofacial applications.

### 2.3 AI optimizes 4D bioprinting processing parameters

Printing temperature, layer height, infill density and scanning trajectories collectively determine the final deformation kinetics and mechanical performance of a smart construct (Samal et al., 2024). The complex and highly non-linear design space inherent in 4D printing makes it challenging for conventional trial-and-error workflows to satisfy multiple, and sometimes conflicting, performance constraints simultaneously (Lu et al., 2026). Bai et al. (2025) proposed a deep reinforcement learning-based printing path optimization algorithm (DRL-PN), embedding thermo-mechanical coupling effects of laser melting into environmental modeling, with composite reward functions guiding directional printing path planning. Numerical simulations and experimental validation demonstrated that the proposed framework improved path generation efficiency by 99.8%, while reducing structural deformation and residual stress by 64.0% and 21.8%, respectively. By formulating additive manufacturing as a sequential decision-making problem optimized for long-term reward maximization, this algorithm can be extended to joint optimization of nozzle switching timing and scanning trajectories in multi-material 4D bioprinting.

### 2.4 AI-mediated inverse design and deformation programming

Multivariate regulation dominates the deformation behavior of smart materials, creating obstacles to forecasting final deformation states. AI, via inverse design frameworks, deep learning optimization and data-driven modeling, can systematically tackle critical bottlenecks including precise deformation programming and personalized anatomical adaptation for 4D-printed implants, providing a computable and predictable technical pathway to advance the clinical translation of dynamic smart implants (Jiao et al., 2025). Chiu et al. (2025) proposed an inverse design pipeline for dual-material 4D bioprinted facial curved surfaces using fully convolutional neural networks (FCNs) trained on over 100,000 nonlinear finite element simulation datasets. The FCN directly predicted 2D printable mesh configurations from target 3D depth images, achieving pixel-level prediction accuracy and intersection over union (IoU) exceeding 90%, validated on complex curved structures including Noh masks. Similarly, Chen et al. (2025) applied FCNs to inverse design and forward prediction of dual-material 4D bioprinted facial shells, proposing a Curve Matrix design strategy that modulates material ratios and rib arc angles to generate diverse deformation modes mimicking protruding nasal subunits. Three FCN architectures were developed for linear matrix inverse design, curve matrix inverse design and forward deformation prediction. Under a multi-task learning framework, the model simultaneously predicted rib material composition and curvature distribution, and transfer learning enabled generalization from simulated datasets to authentic human facial anatomical data. Experimental bioprinted facial masks and nasal thin shells exhibited depth deviation less than 10% of overall dimensions compared with simulation predictions, verifying full-chain feasibility from data-driven inverse design to physical biomanufacturing as reported by Chen et al. (2025).

The full closed-loop workflow integrating smart biomaterial selection, AI optimized design, 4D biomanufacturing and *in vivo* craniomaxillofacial regeneration is systematically summarized in Figure 1. This schematic illustrates the complete technical chain from stimuli-responsive smart 4D biomaterials and multi-algorithm AI design pipeline to physical scaffold fabrication and final reconstructive outcomes, as well as the existing translational obstacles and future developmental directions of this field.

**FIGURE 1:**
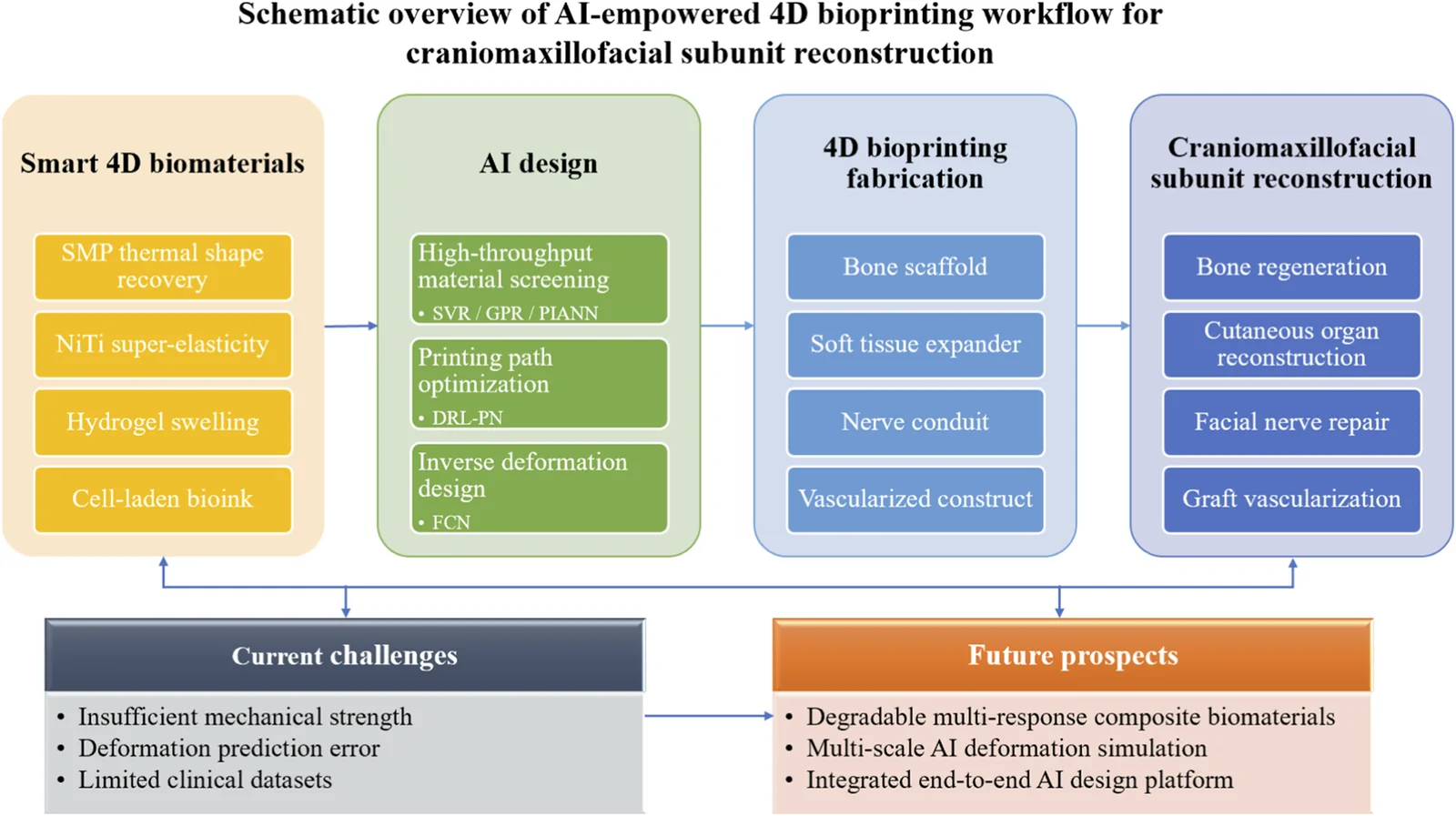
Schematic overview of AI-empowered 4D bioprinting workflow for craniomaxillofacial subunit reconstruction. The workflow is divided into four core modules: smart 4D biomaterials, AI design, 4D bioprinting fabrication and craniomaxillofacial subunit reconstruction. Smart shape-morphing biomaterials including SMP, NiTi alloy, hydrogel and cell-laden bioink act as the material basis. The AI design module integrates SVR/GPR/PIANN for material screening, DRL-PN for printing path optimization and FCN for inverse deformation design. After fabrication of bone scaffolds, soft tissue expanders, nerve conduits and vascularized constructs, *in vivo* implantation achieves bone, ear and facial nerve reconstruction. The figure also summarizes main current challenges and corresponding future development directions of this regenerative strategy.

## 3 Programmable 4D bioprinted constructs for craniomaxillofacial subunit reconstruction

To systematically summarize the material design, deformation mechanisms, experimental outcomes and translational prospects of 4D bioprinted scaffolds for different craniofacial subunit repair scenarios, we organize representative research advances in Table 3.

**TABLE 3:** Programmable 4D bioprinted constructs for craniomaxillofacial subunit reconstruction.

Reconstruction category	Smart material and trigger	Key outcomes	Translational value
Bone defect repair	NIR-SMP; gradient alginate (Ca^2+^); magnetic Fe_3_O_4_-PLLA	NIR-triggered recovery + osteogenesis (Guo et al., 2025); 0.05–0.446 mm^-1^ curvature (Lv et al., 2025); >98% recovery/40 s + immunomodulation (Song et al., 2025)	Minimally invasive repair of irregular cranial/orbital/maxillary defects
Soft tissue and cutaneous organ reconstruction	DLP-swellable hydrogel; AEH-TEs	Skin area ↑2-fold, weight ↑3-fold at 5 days post-implantation (Cao et al., 2025); auricle/breast expanders (Wang et al., 2026)	Personalized auricle, nose, and facial soft tissue reconstruction
Peripheral nerve regeneration	Oriented SMP; wrinkled SMP	Nerve defect repair efficiency ↑60% (Sun et al., 2024); aligned OEC morphology (Jones, 2023)	Facial and trigeminal nerve repair
Vascularized composite tissues	Alginate/methylcellulose; bilayer periosteal matrix	Perfusable T-shaped branched vascular channels (Nain et al., 2024); osteogenesis-angiogenesis coupling (McLoughlin, 2025)	vascularized soft-hard composite systems

AEH-TE, adaptive elastic hydrogel tissue expander; Ca^2+^, calcium ion; DLP, digital light processing; OEC, olfactory ensheathing cell; PLLA, poly (lactic acid); SMP, shape memory polymer.

### 3.1 4D bioprinted scaffolds for craniomaxillofacial bone defect repair

Craniomaxillofacial bone defects feature complex, irregular curved geometries. Conventional static 3D printed rigid implants require large surgical incisions and additional fixation hardware during implantation (Guo et al., 2025). Compressible 4D bioprinted scaffolds can be delivered via minimally invasive approaches and recover pre-designed 3D conformations upon *in vivo* stimulation to fit irregular bone defects, while incorporating osteogenic bioactive components to drive bone regeneration (Song et al., 2025).

Guo et al. (2025) fabricated biomimetic dual-function 4D bioprinted scaffolds with precisely tunable shape transition temperatures ranging from 37 °C to 42 °C via copolymer monomer modulation, enabling mild, safe shape recovery triggered by near-infrared light. The scaffold compresses for minimally invasive delivery and recovers anatomically matched 3D conformations post-implantation. Hydrogel layers loaded with nano-hydroxyapatite@polydopamine-pargyline composites enhance scaffold hydrophilicity and continuously release osteogenic factors to facilitate osteogenic differentiation of stem cells. In rat cranial defect models, the composite scaffold exhibited outstanding anatomical conformity and robust bone regenerative capacity. Lv et al. (2025) developed gradient photocrosslinkable alginate hydrogel 4D bioprinted self-bending scaffolds. Sequential crosslinking and gradient photopolymerization induced spontaneous bending with curvatures ranging from 0.05 to 0.446 mm^-1^ after calcium ion and chitosan treatment, significantly accelerating new bone formation in rodent cranial defect models.

To expand the multifunctional applications of 4D bioprinted bone scaffolds, Song et al. (2025) integrated magnetic responsiveness and immunomodulatory functionality into poly (lactic acid) (PLLA) 4D bioprinted scaffolds loaded with Fe_3_O_4_ nanoparticles, composited with hesperidin-incorporated chitosan hydrogel. The scaffold achieved shape recovery ratios exceeding 98% within 40 s upon external magnetic stimulation, enabling conformation to irregular bone defects. Beyond its shape memory performance, the scaffold exerted immunomodulatory effects through regulation of the NOD-like receptor family pyrin domain containing 3 (NLRP3) inflammasome pathway. Specifically, the Fe_3_O_4_-PLLA/hesperidin-chitosan scaffold suppressed NLRP3 inflammasome activation in macrophages, thereby promoting the phenotypic switch from the pro-inflammatory M1 phenotype toward the anti-inflammatory M2 phenotype. This M2-dominant immune microenvironment further enhanced the osteogenic differentiation of bone marrow mesenchymal stromal cells (BMSCs) via paracrine signaling. The scaffold achieves a synergistic integration of physical shape adaptation and biological immunomodulation, establishing a favorable osteoimmune microenvironment that couples inflammation resolution with endogenous bone regeneration.

### 3.2 4D bioprinting for facial soft tissue and cutaneous organ reconstruction

Repair of facial cutaneous and mucosal defects often requires tissue expanders to generate sufficient autologous graft material. Conventional inflatable expanders achieve homogeneous isotropic swelling and fail to accommodate irregular individual anatomical contours. Cao et al. (2025) reported biocompatible 4D bioprinted hydrogel expanders fabricated via digital light processing (DLP), composed of swellable polymer sheets and non-extensible elastic frameworks. Contact with interstitial fluid induces programmable anisotropic buckling deformation to form pre-designed 3D architectures. Five days post-subcutaneous implantation in rat scalp models, skin area and weight increased 2-fold and 3-fold without local tissue injury. Wang et al. (2026) further translated 4D bioprinted expanders toward precise reconstruction of cutaneous organs including auricles, noses and mammary glands. Polyelectrolyte hydrogel-based adaptive elastic hydrogel tissue expanders (AEH-TEs) were fabricated via DLP 4D bioprinting, directly converting patient-specific 3D scanning anatomical data into personalized complex 3D constructs. In rabbit models, auricle-shaped AEH-TEs expanded autologous skin flaps with anatomical contours highly consistent with native auricles, eliminating trimming requirements before prosthetic implantation. Mammary gland-shaped AEH-TEs expand to average adult breast dimensions with a basal diameter of approximately 10 cm, supporting robust neovascular network formation in expanded subcutaneous tissue. The hydrogel matrix autonomously absorbs hematoma fluid while maintaining stable swelling functionality, preventing postoperative hematoma complications in tissue expansion surgery.

### 3.3 4D bioprinted scaffolds for craniomaxillofacial peripheral nerve regeneration

The craniomaxillofacial region contains dense peripheral nerve networks including facial and trigeminal nerves, whose functional recovery post-injury remains a major bottleneck in reconstructive surgery. Static conventional scaffolds cannot generate spatiotemporally controllable regenerative microenvironments, while dynamically deformable 4D bioprinted scaffolds enable non-invasive remote regulation of neuronal behavior and cell fate, exhibiting promising potential for craniomaxillofacial nerve repair. Sun et al. (2024) fabricated oriented dynamic 4D bioprinted scaffolds from SMPs, integrating programmable microtopography and mechanical stretching stimulation to realize spatiotemporal modulation of neuronal proliferation at early stages and axonal differentiation and elongation in later phases. The scaffold improved repair efficiency of large-segment nerve defects by 60%, with therapeutic efficacy comparable to autologous nerve grafting. Jones, 2023 investigated contact guidance behavior of olfactory ensheathing cells cultured on wrinkled SMP substrates fabricated via programmable 4D bioprinting. Shape recovery of SMP matrices generated surface micro-wrinkles that induced elongated, aligned morphology of olfactory ensheathing cells along wrinkle orientations.

### 3.4 4D bioprinting for fabrication of vascularized composite tissues

Sufficient microvascular networks represent a prerequisite for long-term survival of large-volume craniomaxillofacial composite grafts. Previous studies identified 4D bioprinting, alongside suspension bioprinting and coaxial bioprinting, as cutting-edge biofabrication technologies for generating vascularized oral and maxillofacial tissues, with unique advantages in constructing dynamic vascular networks via programmable morphological deformation (Chen et al., 2023). Nain et al. (2024) developed a nanoengineered alginate/methylcellulose two-component hydrogel system. By adopting 4D printing technology, they successfully fabricated perfusable T-shaped branched vascular channels with antioxidant, anti-inflammatory and antithrombotic activities. Precise prediction of deformation behavior was achieved via computational models, which provides experimental evidence for constructing complex branched vascular networks using 4D printing. Although this study focused on the fabrication of *in vitro* vascular models, its design concepts and deformation regulation strategies offer crucial references for the *in vivo* construction of craniofacial microvascular networks in the future. In the field of craniofacial tissue engineering, Fisher’s research group at the University of Maryland developed dual-layer 4D bioprinted periosteal composite constructs, consisting of an inner osteoprogenitor-laden layer and an outer dense collagen matrix containing integrated nerve and microvascular networks (McLoughlin, 2025). The construct aims to generate synchronized osteogenesis and angiogenesis *in vivo*, representing a critical advancement of 4D bioprinting from monolithic bone scaffolds to integrated vascularized soft-hard composite systems.

## 4 Current challenges

### 4.1 Limitations of smart biomaterials

Stimuli-responsive smart materials form the functional foundation of 4D bioprinting; however, existing material systems exhibit trade-offs between mechanical performance, biocompatibility and clinically compatible deformation triggers. Load-bearing craniomaxillofacial regions such as the mandible sustain occlusal forces of 300–500 N, while the compressive moduli of most SMPs remain below 100 MPa, insufficient to maintain long-term mechanical stability after programmed deformation (Ferrer et al., 2024). Thermally responsive SMPs require glass transition temperatures (Tg) slightly above physiological temperature (37 °C) to avoid unintended *in vivo* deformation (Aldana et al., 2024). Precise modulation of Tg within a safe therapeutic window (37 °C–42 °C) remains challenging: excessively high Tg induces thermal injury to adjacent soft tissue, while low Tg triggers unplanned deformation driven by local inflammatory hyperthermia. Long-term *in vivo* stability and precisely tunable drug release kinetics of hydrogel matrices under complex physiological environments enriched with enzymes, proteins and dynamic mechanical loading require further systematic validation (Chelu et al., 2026).

### 4.2 Insufficient precision and reproducibility of programmable deformation

Clinical efficacy of 4D bioprinted implants relies on predictable, reproducible deformation kinetics. Most existing research validates deformation performance under idealized laboratory conditions, while complex physiological microenvironments introduce substantial prediction uncertainty. The deformation trajectories of shape-memory polymers are governed by coupled interactions among material constitutive parameters, geometric configurations, stimulus magnitudes and loading modalities. Even for multi-stage shape-memory architectures exemplified by triple-shape-memory scaffolds, extensive experimental optimization is required to realize coordinated modulation of deformation triggering windows, shape recovery ratios and response times across different stages (Sabahi et al., 2026). Applications of AI in this field remain in their nascent phase. More fundamentally, current AI frameworks for predicting 4D printing deformation are predominantly trained using finite element simulation datasets rather than physical experimental measurements. The persistent “virtual-real gap” between numerical simulation and physical reality may trigger model failure during *in vivo* translation.

### 4.3 Limitations of clinical applicability of AI models

AI- empowered 4D printing is fundamentally reliant on high-quality multimodal datasets with standardized annotations and broad coverage of diverse clinical variations, yet such foundational resources remain severely underdeveloped for craniomaxillofacial reconstruction. Craniomaxillofacial defects are induced by heterogeneous causes and marked by substantial interpatient morphological heterogeneity. Existing public datasets are plagued by limited sample sizes, inconsistent annotation standards and inadequate coverage of defect subtypes, inevitably leading to pronounced performance deterioration of AI models during multi-center and multi-population external validation (Wang et al., 2025). Furthermore, although deep reinforcement learning algorithms have achieved tentative advances in optimizing nozzle switching sequences, crosslinking density gradients and spatial configuration for multi-material 4D printing, significant precision loss and inherent batch-to-batch variability persist in parameter translation between computational outputs and physical printing hardware (Tripathi et al., 2025). Moreover, the majority of investigations into AI-assisted material screening and inverse design remain confined to proof-of-concept explorations. To date, researchers lack a systematic framework for robustness evaluation and uncertainty quantification, creating a critical translational gap between simulation-trained models and practical clinical decision-support systems deployed in real-world settings.

## 5 Future prospects

Current applications of smart materials in craniomaxillofacial reconstruction are restricted by inherent performance limitations, inaccurate deformation control, and fragmented technical systems. Artificial intelligence (AI) holds great potential to facilitate the comprehensive optimization and clinical transformation of relevant technologies. In material research and development, data-driven strategies and AI inverse design frameworks can overcome the limitations of conventional trial-and-error methods. Composite smart materials with balanced mechanical properties, multi-stimulus responsiveness, and excellent biocompatibility can be rationally developed to precisely satisfy the differentiated repair requirements of diverse anatomical regions in the craniomaxillofacial system. In terms of technical accuracy, the establishment of multi-scale coupled simulation models and intraoperative AI closed-loop feedback correction systems can eliminate deformation errors induced by individual anatomical differences and material batch variations, thereby improving the *in vivo* adaptability, accuracy and stability of 4D-printed implants. Furthermore, combining AI high-throughput analysis with multi-dimensional experimental models enables the construction of a standardized evaluation system for the *in vivo* safety and immune compatibility of smart materials. Precise microenvironment regulation achieves the synergistic integration of deformation adaptation, tissue regeneration and immune homeostasis, which consolidates the safety guarantee for clinical popularization. In the long term, the full-process AI intelligent design platform integrating medical image analysis, material screening, deformation simulation and printing optimization represents the core developmental trend of this field. This platform is expected to realize the automatic and intelligent customized fabrication of craniomaxillofacial prostheses, resolve critical clinical challenges including allogeneic donor scarcity and immune rejection, and promote the paradigm shift of craniomaxillofacial reconstructive surgery from traditional allogeneic tissue replacement to AI-enabled autologous regenerative reconstruction.

Table 4 consolidates the core limitations across smart materials, programmable deformation, and AI clinical translatability, alongside corresponding future directions to bridge the translational gap from bench to bedside.

**TABLE 4:** Current challenges and future prospects of AI-empowered 4D bioprinting.

Challenge domain	Current limitations	Future prospects and solution directions	References
Smart biomaterials	Trade-offs: strength vs. biocompatibility vs. triggers; SMP modulus <100 MPa (mandible 300–500 N); Tg tuning 37 °C–42 °C difficult; hydrogel stability/drug-release kinetics lack systematic validation	AI inverse design for balanced composites; region-specific formulations; standardized *in vivo* safety/immune evaluation	Ferrer et al., 2024; Aldana et al., 2024; Chelu et al., 2026
Programmable deformation	Lab-only validation; multi-stage SMP needs extensive experimental optimization; AI models trained on FEA (virtual–real gap)	Multi-scale simulation + intraoperative AI feedback; correct individual/batch variations; improve *in vivo* adaptability/stability	Sabahi et al., 2026; Bond et al., 2023
AI model clinical translatability	Lack of high-quality multimodal datasets; small samples and inconsistent annotations; computational-to-physical translation loss; most studies proof-of-concept only	Standardized multi-center datasets; robustness/uncertainty quantification; bridge simulation-to-clinical systems	Wang et al., 2025; Tripathi et al., 2025
Integrated platform and paradigm shift	Fragmented systems; reliance on allogeneic tissue donors and lifelong immunosuppression	Full-process AI platform (image analysis → screening → simulation → printing); shift to autologous regenerative reconstruction	None

AI, artificial intelligence; FEA, finite element analysis; SMP, shape memory polymer; Tg, glass transition temperature.

## 6 Conclusion

This review comprehensively summarizes cutting-edge advances of AI-empowered 4D bioprinting for craniomaxillofacial subunit reconstruction, revealing transformative synergies across bone defect repair, soft tissue reconstruction, peripheral nerve regeneration and vascularized composite tissue fabrication. Incorporation of time-dependent programmable deformation enables minimally invasive delivery of 4D bioprinted scaffolds that spontaneously conform to irregular defect contours and guide endogenous tissue regeneration, fundamentally overcoming passive anatomical matching limitations of static 3D printed implants. Artificial intelligence reshapes the entire research workflow of 4D bioprinting from high-throughput smart material screening and manufacturing trajectory optimization to precise deformation programming. Nevertheless, this field confronts persistent challenges including insufficient mechanical strength of existing smart materials, limited accuracy of deformation prediction, and scarcity of clinically translatable human data. Deep integration of artificial intelligence and 4D bioprinting via all-in-one intelligent design platforms holds great promise to realize full craniomaxillofacial subunit regeneration without allogeneic tissue donors or lifelong immunosuppression in the future.
